# A meiotic mystery: How sister kinetochores avoid being pulled in opposite directions during the first division

**DOI:** 10.1002/bies.201500006

**Published:** 2015-04-15

**Authors:** Kim Nasmyth

**Affiliations:** Department of Biochemistry, Oxford UniversityOxford, UK

**Keywords:** cohesion, co-orientation, kinetochore, meiosis, monopolin

## Abstract

We now take for granted that despite the disproportionate contribution of females to initial growth of their progeny, there is little or no asymmetry in the contribution of males and females to the eventual character of their shared offspring. In fact, this key insight was only established towards the end of the eighteenth century by Joseph Koelreuter's pioneering plant breeding experiments. If males and females supply equal amounts of hereditary material, then the latter must double each time an embryo is conceived. How then does the amount of this mysterious stuff not multiply exponentially from generation to generation? A compensatory mechanism for diluting the hereditary material must exist, one that ensures that if each parent contributes one half, each grandparent contributes a quarter, and each great grandparent merely an eighth. An important piece of the puzzle of how hereditary material is diluted at each generation has been elucidated over the past ten years.

## Introduction

It is, of course, now widely appreciated that the replication and segregation of DNA molecules each time a cell divides forms the basis for most hereditary phenomena. During mitotic divisions, sister DNAs produced during S phase are segregated to opposite poles of the cell, with the result that daughters inherit exactly the same set as their mothers. Because of their immense length, chromosomal DNAs are packaged into cylindrical structures (chromatids) that fit into each half of the dividing cell and have an elasticity that maintains their shape when dragged around the cell by microtubules. Remarkably, sister DNAs are not jumbled together but “condensed” into individual sister chromatids that remain tenuously held together along an inter-chromatid axis (sister chromatid cohesion). These two features are mediated by a pair of multi-subunit complexes called condensin [1] and cohesin [2]. Related complexes exist in eu- and arche-bacteria, implying an ancient origin.

At the heart of these complexes is a pair of rod-shaped Smc proteins possessing ABC-like ATPase and dimerization domains at opposite ends of 50 nm long anti-parallel intra-molecular coiled coils. Bacterial Smcs form V-shaped homo-dimers, while their eukaryotic counterparts, condensin and cohesin, contain Smc2/Smc4 and Smc1/Smc3 hetero-dimers, respectively. In all cases, the two ATPase heads of Smc dimers are inter-connected in an asymmetric manner by kleisin subunits, whose N- and C-terminal domains bind, respectively, to the coiled coil emerging from one ATPase and the base of the ATPase of its partner, thereby generating a gigantic tripartite ring [3–5]. Cohesin rings formed by the pairwise interactions between Smc1, Smc3, and an α-kleisin, entrap sister DNAs in living yeast cells, and this presumably is the mechanism by which they hold sister chromatids together. Whether condensin holds together the individual DNAs of a single chromatid using a similar topological mechanism is unknown. What is clear is that disjunction of sister chromatids at the metaphase to anaphase transition is triggered by proteolytic cleavage of cohesin's kleisin subunit by separase, which opens the ring and releases sister DNAs from their embrace [6]. Because most, if not all, cohesion is created during DNA replication, separase must remain inactive from S phase until the onset of anaphase, a process mediated by an inhibitory chaperone called securin and phosphorylation by cyclin B/Cdk1 [7].

One of the distinguishing features of chromosome segregation in eukaryotic cells is their use of microtubules (MTs), which attach to specialized regions of the chromosome called centromeres. The network of proteins that connect centromeres and microtubules is called the kinetochore. It has three different layers: an inner layer associated with DNA, an outer layer associated with microtubules, and a central layer that connects the two. Centromeres are usually composed of repetitive DNAs that bind site-specific DNA binding proteins, which in turn promote assembly of nucleosomes whose histone H3 is replaced by a variant called CENP-A. A crucial part of the central layer is the MIND complex, composed of four proteins, one of which is called Dsn1 [8]. Importantly, in mitotic cells, distinct kinetochores form on each sister chromatid, a phenomenon facilitated by their individualization into separate chromatin domains with the help of condensin.

Microtubules must pull sister kinetochores in opposite directions. This comes about because connections between microtubules and outer kinetochore proteins are stabilized by tension [9,10], which is only created if cohesion between sister chromatids resists traction of sister kinetochores to opposite poles of the cell [11]. Tension sufficient to stabilize kinetochore microtubules is not created when sisters are erroneously pulled in the same direction, which presents an opportunity to try again. The only stable state of this system is the attachment of every pair of sister kinetochores to microtubules pulling in opposite directions, which is known as bi-orientation. This error correction system takes time, which is provided by a regulatory network called the spindle assembly checkpoint (SAC). Kinetochores that have not yet bi-oriented and not come under tension create an inhibitor that blocks activation of the anaphase promoting complex or cyclosome (APC/C), a gigantic ubiquitin protein ligase whose destruction of securin and cyclin B unleashes separase [12].

Centromeres have another important function: to confer sister chromatid cohesion capable of resisting spindle forces. In *S. cerevisiae*, especially high levels are generated in a 50 kb interval surrounding their point centromeres, a process, catalyzed by cohesin's Scc2/4 loading complex together with proteins associated with the inner centromere COMA complex [13]. How other eukaryotes promote cohesion in the vicinity of their centromeres is not known. Better understood, however, is the mechanism by which cohesion, once established, is selectively maintained. In most eukaryotic cells but not in yeast, cohesin dissociates from chromosome arms during prophase due to a releasing activity associated with its Wapl [14], Pds5, and SA/Scc3 subunits. Release is mediated by transient dissociation of α-kleisin's N-terminal domain from Smc3, creating an “exit” gate through which entrapped DNAs can escape the cohesin ring. In yeast cells, releasing activity is shut off during S phase through modification by the Eco1 acetyl transferase of a pair of highly conserved lysine residues on Smc3's ATPase head adjacent to where α-kleisin's N-terminal domain binds [15]. In animal cells, neutralization of releasing activity requires in addition the recruitment of sororin. As cells enter M phase, sororin—together with other cohesin subunits—is phosphorylated by mitosis-specific kinases such as Cdk1 and Polo-like kinase (PLK). This inactivates sororin and thereby activates the releasing activity that triggers cohesin's dissociation from chromosomes. This process is known as the prophase pathway, to distinguish it from separase mediated release [16].

The prophase pathway is capable of destroying all sister chromatid cohesion, and as such it represents a mortal threat to mitotic chromosome segregation. Crucially, one of the key functions of centromeres is to recruit a set of proteins known as shugoshins [17]. Key features of shugoshins are a C-terminal domain that confers recruitment to centromeres, a central domain that binds SA/Scc3 [18], and an N-terminal domain that forms an inter-molecular parallel coiled coil that mediates dimerization and provides a binding platform for phosphatase PP2A containing its B’ regulatory subunit [19]. By recruiting PP2A to centromeres and simultaneously binding SA/Scc3, shugoshins prevent phosphorylation of sororin and SA/Scc3 in the vicinity of centromeres, thereby protecting cohesin from the prophase pathway. Mammalian cells express two shugoshins, Sgol1 and Sgol2. The former is essential for protecting centromeric cohesion from the prophase pathway during mitosis [20] while the latter protects it from separase during meiosis [21]. The mechanism by which shugoshins are recruited to centromeres remains poorly understood. The process depends on kinase activity of Bub1—which is also a central component of the SAC—and is thought to involve phosphorylation of S121 on histone H2A [22]. However, this residue cannot be Bub1's sole (if at all) target, because recruitment of Sgo1 to centromeres in yeast remains Bub1-dependent even when S121 is replaced by aspartic acid [23].

By ensuring traction of sister chromatids to opposite sides of the cell prior to its division, the complex network of chromosomal proteins described above serves to maintain chromosome numbers during vegetative cell proliferation, during which each round of DNA replication is succeeded by chromosome segregation and vice versa. The APC/C has a central role in ensuring that S and M phases alternate. By destroying mitotic cyclins and geminin along with securin at the onset of anaphase, the APC/C not only triggers chromosome segregation, but also creates a state permissive to the assembly of pre-replication complexes at replication origins, which only fire when Cdk1 is re-activated during the next cycle [24,25].

## Vive la difference!

### Changes needed to convert mitosis to meiosis

How is this inherently conservative system of DNA replication, sister chromatid cohesion, kinetochore bi-orientation, and sister chromatid disjunction corrupted with the view to reducing the number of each chromosome from two to one, as occurs during meiosis? One might imagine this to be a rather simple matter. A priori, all that is needed is a system that pairs up unreplicated homologous chromosomes. If so, the same mechanism used during mitosis could disjoin homologs instead of sisters in what would be a *reductional* instead of an *equational* division. Contrary to expectation, the first meiotic division is preceded by chromatid (DNA) replication and as a consequence two peculiar divisions (meiosis I and II) are required to halve chromosome numbers.

**Figure 1:**
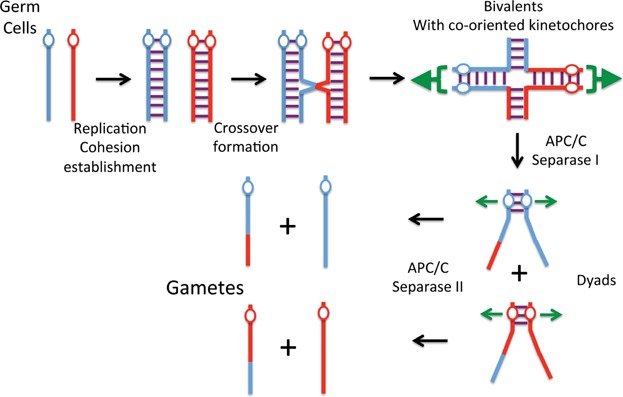
The logic behind halving chromosome numbers through two meiotic divisions.

The mitotic program must be altered in several ways to achieve this goal. The first is manifest after replication when a single reciprocal recombination between non-sister homologous chromatids (a crossover) creates a totally new type of chromosome, namely a bivalent containing four chromatids: two parental and two recombinant [26]. Crucially, recombination leaves cohesion created during DNA replication intact, except in the immediate vicinity of crossovers, and it is this cohesion that holds together the bivalent's four chromatids (Fig. 1). Though a single crossover between homologous chromosomes is sufficient to create a bivalent, multiple crossovers occur in many organisms.

In yeast [27,28] and mammals [29], meiotic sister chromatid cohesion is mediated by cohesin containing a meiosis-specific α-kleisin subunit called Rec8, which has a number of special properties. Two are crucial for creating and maintaining bivalents. Rec8 ensures that double strand breaks initiating recombination are repaired by non-sister DNAs [30]. It also seems largely immune to the prophase pathway, which in animal cells would otherwise destroy sister chromatid cohesion along arms. As a result, chromosomes enter the first meiotic M phase as bivalents possessing four centromeres. If each built its own kinetochore, then there would be more than one way of creating tension. How do cells deal with this potentially lethal ambiguity? They manage to do so because of the third key feature of meiosis, namely that co-orientation of sister kinetochores (i.e. attached to MTs emanating from the same pole) is systematically favored over bi-orientation (i.e. attached to MTs emanating from different poles) [31]. Something prevents bi-orientation, and bivalents therefore possess only two functional kinetochores, a maternal and a paternal one, which are then pulled in opposite directions [32].

As in mitosis, the first meiotic division is triggered by separase, whose cleavage of Rec8 along chromosome arms destroys the cohesion holding bivalents together, and triggers disjunction to opposite poles of pairs of chromatids attached to maternal and paternal kinetochores, respectively [33]. Crucially, these “dyads” remain cohesed at their centromeres because PP2A associated with shugoshins [19] counteracts Rec8's phosphorylation by Cdc7 and casein kinase delta (Hrr25 in yeast), which is a precondition for its cleavage [34,35]. Protection of centromeric Rec8 followed by loss of co-orientation and no DNA replication between divisions ensures that dyads bi-orient in a conventional manner during meiosis II and sister centromeres disjoin upon a second round of separase activation, creating gametes containing only a single copy of the genome (Fig. 1).

## Meiosis

### Why is it so elaborate?

Why the chromosome reduction required for sexual reproduction involves two divisions and is so much more complicated than mitosis has long been a mystery. One can envisage two sorts of answers. The first is physiological. Recombination is a simple mechanism for joining homologs together, but it can only do so if sister chromatids are held together by cohesion, which must be created by a round of DNA replication. According to this scenario, two divisions are necessary because replication precedes meiosis. The second explanation is evolutionary. If one assumes that we reproduce sexually in order to eliminate deleterious mutations [36] or to create variety amongst offspring—and that recombination is therefore a fundamental aspect of meiosis—then the question arises as to why recombination takes place after DNA replication and not before it. Note that in this case, homologs would have to pair using a mechanism that did not involve sister chromatid cohesion, which is frequently the case in the heterogametic sex of Lepidoptera. It has been suggested that recombination after replication protects gametes from sister killer genes and other forms of meiotic drive [37]. Interestingly, certain organisms with holo-centric centromeres (i.e. those distributed throughout the chromosome) reduce chromosome numbers during meiosis using a very different scheme involving the segregation of sister centromeres at the first and disjunction of homologs only at the second meiotic division [38]. We may never know why meiosis functions as it does because eukaryotes with a more rudimentary mechanism possibly present in eukaryotic ancestors have never been described and may not have survived.

### Monopolin confers co-orientation in *S. cerevisiae*

Of the five hallmarks of meiosis, co-orientation is the least understood. Moreover, no universal mechanism has so far emerged. Best characterized is *S. cerevisiae*, where a multi-subunit complex called monopolin confers this property [39]. Three of its subunits, namely Csm1, Lrs4, and the casein kinase I Hrr25 are present in mitotic cells as well as meiotic ones, but the fourth, Mam1, is only expressed during the first meiotic division [40]. In mitotically dividing cells, Csm1 and Lrs4 form a complex that resides in the nucleolus [41], where it recruits condensin to replication fork barriers within each rDNA repeat [42]. This function helps prevent unequal crossing over, and facilitates the individualization of sister nucleoli necessary for efficient disjunction of sister rDNAs at anaphase. Csm1/Lrs4 also resides in the nucleolus during the early phases of meiosis, but is released from this location upon accumulation of the Cdc5 polo-like kinase (PLK) during prophase [43]. Csm1/Lrs4 then binds to Mam1, which in turn recruits the Hrr25 casein kinase delta, thereby forming a holo-monopolin complex.

### Multiple meiotic roles of the Cdc5 Polo-like kinase (PLK)

Cdc5 has two additional functions promoting co-orientation. First, it triggers resolution of double holliday junctions created through the repair of Spo11-induced double strand breaks, to produce reciprocal crossovers, thereby generating bivalent chromosomes [44]. Second, its Polo Box Domain (PBD) binds a conserved STSTP motif within a meiosis-specific protein called Spo13 [45]. The resulting Spo13/Cdc5 complex associates with another kinase Dbf4/Cdc7 and together they promote phosphorylation of Lrs4 [46,47]. Lrs4 fails to be phosphorylated and holo-monopolin complexes fail to associate with kinetochores in *spo13* mutants with a mutated STSTP motif. Interestingly, Cdc5 also has a role in promoting protection of centromeric Rec8 [34], presumably by regulating in some way the activity of shugoshin-PP2A complexes. In meiotic cells lacking Cdc5, Dbf4/Cdc7, Spo13, or any of monopolin's four subunits, sister kinetochores bi-orient instead of co-orienting. However, because protection of centromeric Rec8 is only partly (if at all) affected by this defect [23], separase fails to trigger sister centromere disjunction, and the first meiotic division fails to take place [40]. Spo13 is destroyed by the APC/C at the onset of anaphase I, an event that could contribute to the lack of monopolin activity after meiosis I. However, this cannot be the sole mechanism, because a non-degradable version of Spo13 does not in any way compromise chromosome segregation during meiosis II [48].

### What is monopolin's mechanism?

How does monopolin force sister kinetochores to act as single unit? Crystal structures show that Csm1 forms a rod-shaped homodimeric protein with an N-terminal coiled coil and a C-terminal globular domain. Lrs4 also dimerizes to form a short coiled coil, which through interacting with two different Csm1 dimers creates a V-shaped hexameric complex containing four molecules of Csm1 and two molecules of Lrs4 [49]. C-terminal domains of Mam1 wrap around the globular domains of each Csm1 dimer while more N-terminal sequences bind a single molecule of Hrr25 [50]. Mam1 therefore links each head at the vertices of the V-shaped Csm1/Lrs4 hexamers to Hrr25. Intriguingly, Csm1 is structurally related to the Spc24 and Spc25 subunits of the outer kinetochore Ndc80 complex. In contrast to Csm1, which forms homodimers, Spc24 and Spc25 form heterodimers whose globular C-terminal domains connect the Ndc80 complex to proteins within central layer such as the MIND complex. Might Csm1 have a similar function? Remarkably, a small hydrophobic patch on Csm1's globular domain opposite its coiled coil—and highly conserved amongst fungal orthologs—is required for interaction with the N-terminal domain (NTD) of the MIND complex's Dsn1 subunit [49,51]. Crucially, this patch as well as the Dsn1's N-terminal Csm1 interacting domain (CID) is necessary for monopolin's recruitment to kinetochores as well as for co-orientation.

**Figure 2:**
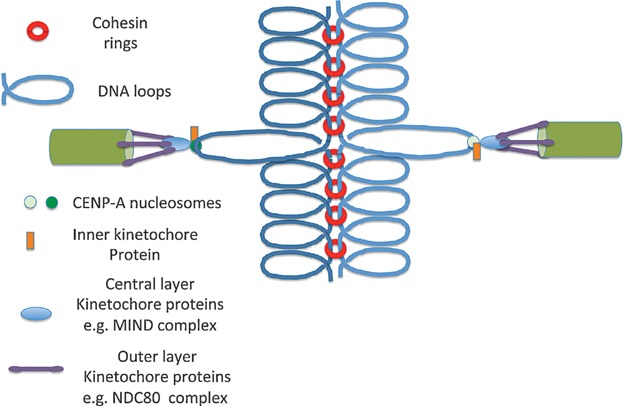
Bi-orientation of sister kinetochores during mitosis in *S. cerevisiae*, where each kinetochore contains only a single CenPA nuclesome and attaches to a single microtubule. Note that due to traction by microtubules, DNA loops attached to kinetochores are extended and sister kinetochores are partially pulled towards opposite poles.

**Figure 3:**
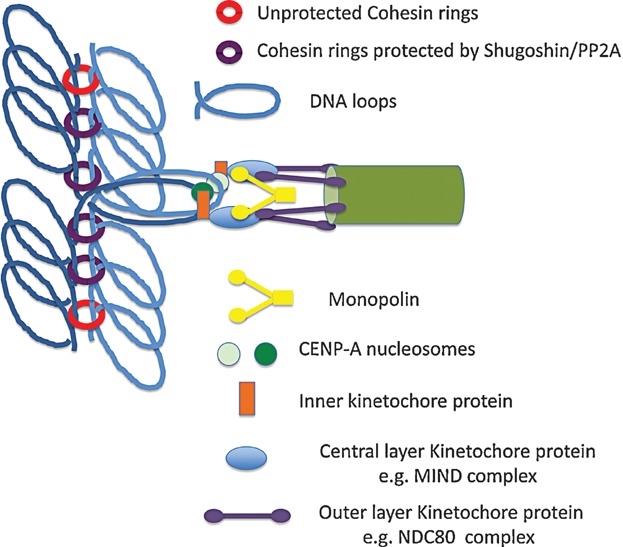
Co-orientation of sister kinetochores during meiosis I in *S. cerevisiae*. Monopolin, which interacts with Dsn1 within the central layer MIND complex, is essential for co-orientation. Whether it functions by cross-linking MIND complexes from sister kinetochores together as shown here is not yet known. It is also not known whether there exists an extra population of cohesin in the vicinity of the inner kinetochore as proposed for *S. pombe* (see Fig. 5).

The finding that monopolin contains two sites for binding Dsn1's CID at each of the two vertices of its V-shaped Csm1/Lrs4 subcomplex suggests that it might mediate co-orientation by crosslinking MIND complexes associated with different sister kinetochores, thereby forcing them to act as a single entity (compare Figs. 2 and 3). Even if broadly correct, this model remains incomplete as it does not explain how monopolin distinguishes MIND complexes of different kinetochores from the 6–8 copies known to be present within each kinetochore. It also does not explain how monopolin avoids merely recruiting more soluble MIND complexes to kinetochores. It is equally plausible that the V-shaped Csm1/Lrs4 complex is not in fact a cross-linking device per se but merely the mechanism responsible for targeting Hrr25 to kinetochores and that co-orientation arises from a signaling network involving Hrr25's kinase activity. It is even conceivable that both of these models are correct and that monopolin does indeed act as a MIND complex cross-linker while its kinase activity modifies other aspects of kinetochore proteins.

Evidence that monopolin acts on kinetochore proteins directly (as opposed to the centromeric DNA underlying them) has come from the ability to isolate intact kinetochores lacking any DNA from yeast cells. These bind and track along microtubules in vitro and even form the “end on” attachments characteristic of bi-oriented kinetochores in vivo [52]. Kinetochores isolated from meiosis I cells are larger and have more stable associations with microtubules than those isolated from mitotic cells, meiosis II cells, or meiosis I *mam1* mutants. Remarkably, kinetochores isolated from mitotic cells can be converted to ones with meiosis I characteristics by the simple addition of holo-monopolin complexes, but only if Dsn1's CID is present [53]. The greater strength of meiotic kinetochores conferred by monopolin could arise from fusing sister kinetochores together. This is not the only explanation and even if true, it does not explain how monopolin specifically fuses sister kinetochores together and avoids merely clustering together multiple kinetochores from different chromosomes.

### Inconsistencies awaiting resolution

Though impressive, several aspects of this work require further resolution. The monopolin complexes used in the strengthening experiments contained an inactive Hrr25 kinase, which stabilizes the recombinant complexes [50] but inactivates them fully in vivo [39]. This raises the possibility that the “add back” experiments are subject to an in vitro artifact. If not, they imply that the complex has a physiologically relevant activity even in the absence of kinase activity, something that had not been revealed merely by in vivo observations. Another observation requiring explanation is that the meiotic kinetochore preparations appear to contain very little if any monopolin, which is difficult to reconcile with the notion that monopolin physically crosslinks MIND complexes together. There are three possible explanations for this. Either very little monopolin is necessary to confer a meiotic property to kinetochores (something that would be easier to comprehend were its kinase activity involved), or the meiotic property can be maintained during a purification procedure that removes most monopolin, or lastly that the kinetochores studied in these experiments are not in a truly meiotic state. The finding that monopolin can strengthen mitotic kinetochores in vitro fits uneasily with the notion that monopolin is required to build but not maintain meiotic kinetochores. It would therefore seem prudent to conclude that we still do not yet fully understand monopolin's molecular mechanism and whether it really acts as a kinetochore cross-linker. Crucial will be to understand the role of its Hrr25's kinase activity.

## Are co-orientation mechanisms conserved?

### Monopolin is not conserved

#### Is modulation of kinetochore proteins a universal feature of co-orientation?

The finding—using RNAi—that a reduction in the levels of another MIND subunit Mis12 causes frequent bi-orientation during meiosis I in maize suggests that it might be, as is the observation that Mis12 forms a continuous plate on top of a pair of distinguishable sets of inner kinetochore proteins [54]. However, Mam1 is poorly conserved even among fungi, as is Dsn1's CID. Though Dsn1 is found in almost all sexually reproducing eukaryotes besides kinetoplastids, its N-terminal CID is only present in yeast species closely related to *S. cerevisiae*. Indeed, its phylogenetic distribution is similar—if not identical—to that of Mam1, and neither can be identified in more distantly related ascomycetes including *Schizosaccharomyces* *pombe* [51]. Csm1 and Lrs4 in contrast are widely distributed among fungi, but their orthologs in *S. pombe* are required for avoiding merotelic attachments during mitosis [55] and not for co-orientation during meiosis I. Moreover, there is evidence that the role of the Csm1/Lrs4 complex in mitotic chromosome segregation in both *S. pombe* [56] and *Candida albicans* [57] involves recruitment of condensin to centromeres, in other words it appears to use a mechanism related to its function at rDNA in *S. cerevisiae*. The implication is that the involvement of Csm1/Lrs4 in co-orientation at meiosis I in budding yeasts may be a derived not an ancient function. This does not necessarily mean that the mechanism revealed by analysis of monopolin in *S. cerevisiae* will not apply to other eukaryotes; merely that the molecules performing any such putative function are insufficiently conserved to be recognized by bioinformatics.

#### Does cohesin containing Rec8 mediate co-orientation in *S. pombe*?

**Figure 4:**
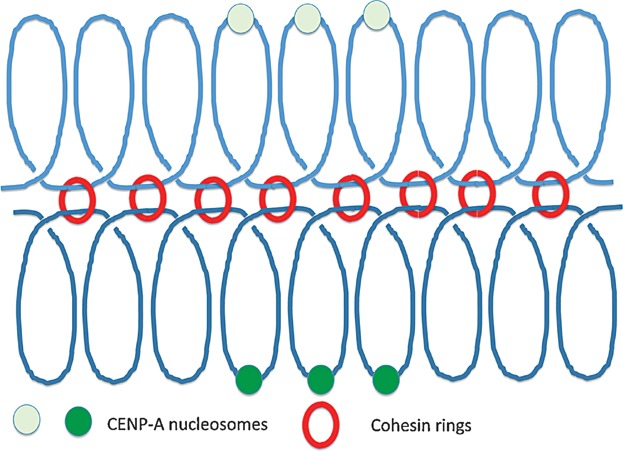
Bi-orientation of sister kinetochores during mitosis in *S. pombe* and possibly in other eukaryotes. Note that each kinetochore has multiple CenpA nucleosomes. CenpA nucleosomes but not other kinetochore proteins are shown.

**Figure 5:**
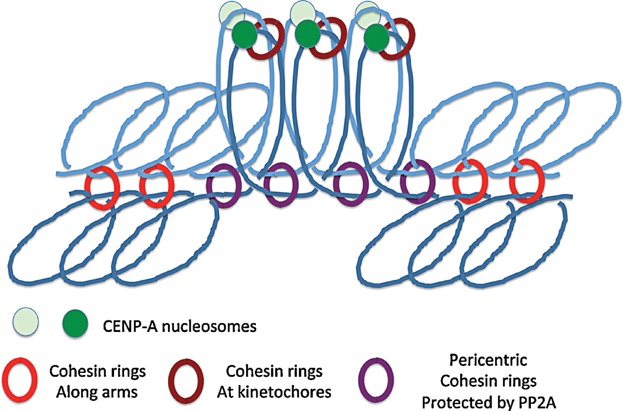
Co-orientation of sister kinetochores during meiosis I in *S. pombe* and possibly in other eukaryotes. It has been proposed that cohesin rings connecting sister DNAs in the vicinity of CenpA nucleosomes contributes to co-orientation instead or as well as hitherto unidentified Monopolin activities that may inter-connect sister kinetochore proteins as proposed for *S. cerevisiae*. Note that according to this model, there are three populations of chromosomal cohesin rings: those along arms that hold bivalents together (see Fig. 1), those holding sister DNAs together in peri-centric regions, and those holding sister kinetchores together in the vicinity of CenpA nucleosomes. Only the peri-centric cohesin rings would be protected from separase by shughoshin/PP2A complexes at the first meiotic division and it is this population that holds dyads together during meiosis II (see Fig. 1).

If monopolin does not mediate co-orientation outside of a small budding yeast clade, then what does? One candidate is Rec8-containing cohesin. The key observation was the finding that *rec8* mutants in *S. pombe* undergo a fully equational division at meiosis I [58]. To understand this, one needs to know that in *S. pombe*, the mitotic version of Rec8, namely Rad21, also contributes to cohesion during meiosis I. Crucially, it fails to do so at core centromeres, which as a consequence are no longer held together, and therefore bi-orient not co-orient. Because Rad21-containing cohesin cannot be protected by shugoshins, bi-oriented kinetochores in *rec8* mutants are disjoined to opposite poles by separase at meiosis I. It has been proposed that Rec8, but not Rad21, has the ability to form cohesion between sister chromatids in the vicinity of inner kinetochore proteins, thereby binding sister DNAs in this region of the chromosome (the core centromere) closer together than would be the case during mitosis [59]. In other words, sister DNA, not sister kinetochore protein cross-linking, mediates co-orientation in *S. pombe* (Compare Figs. 4 and 5).

#### Does the presence/absence of Rec8 cohesion distinguish meiosis II from meiosis I?

To address whether Rec8 binds sister core centromeres during meiosis I but not meiosis II, site-specific recombination was used to loop out core centromeres in cells arrested either in meiosis I, by a *mei4* mutation, or in meiosis II by a *mes1* mutation. Cohesion of the resulting circular DNAs was then measured cytologically using operator arrays bound by repressor proteins fused to GFP. The finding that sister GFP signals associated with circular DNAs were cohesed when created in *mei4* mutants but not in *mes1* mutants is consistent with the hypothesis [60]. However, the experiments failed to document carefully the physical state of the DNAs when excised, and the comparison between meiosis I and II cells has a confounding variable, namely the use of different mutations to arrest the cells. It cannot therefore be excluded that the *mei4* or *mes1* mutation, rather than meiosis I versus meiosis II, was responsible for the difference. The finding that cohesion at core centromeres in *mei4* arrested meiosis I cells, measured using the looping out assay, was dependent on Moa1, a meiosis-specific protein required for co-orientation in cells lacking chiasmata but not in wild type [61], mitigates though does not completely exonerate this flaw. Unlike the monopolin complex in *S. cerevisiae*, Moa1 is not exclusively concerned with co-orientation, and its elimination has rather pleiotropic effects during meiosis I. In addition to the controls mentioned above, the notion that Rec8-containing cohesin mediates co-orientation in *S. pombe* would benefit from a more rigorous analysis of the distribution of Rec8 and Rad21 around centromeres in mitosis, meiosis I, and meiosis II cells using modern genomic techniques. The distribution of acetylated Smc3, which marks complexes exclusively involved in cohesion, would be particularly revealing.

That sister chromatid cohesion may have a more universal role in co-orientation is consistent with the finding that mutations affecting cohesin's kleisin subunits cause bi-orientation of sister kinetochores during meiosis I in plants [62,63]. However, a dependence on Rec8 does not necessarily imply that a special type of cohesion at core centromeres is the mechanism responsible. Cohesion in the vicinity of centromeres is clearly a pre-condition for co-orientation but is it sufficient? Currently lacking are the molecular mechanisms responsible for creating this special type of cohesion and how it actually mediates co-orientation. Another major question concerns when during meiosis I core centromere cohesion is established. This appears to take place during DNA replication in a manner dependent on the Eso1 acetyl transferase [59,64] and yet Moa1 functions even when expressed long after meiotic S phase. If Moa1 acts by promoting core centromere cohesion, then it must do so by maintaining such a structure, not in establishing it in the first place. Whether Moa1 really acts in this manner and if so how is currently not known. Lastly, how is core centromere cohesion destroyed at the onset of anaphase? Is this due to Moa1's destruction after anaphase I, a process reminiscent of Spo13's destruction at the hands of the APC/C in *S. cereviase*, or due to cleavage by separase of the cohesin rings responsible?

### The role of chaperones of Polo-like kinases (PLKs)

Though the mechanism proposed for co-orientation in *S. pombe* appears very different from that elucidated in *S. cerevisiae*, there is one striking similarity: Despite the lack of any sequence conservation, Spo13 and Moa1 share a number of features. They are both poorly conserved meiosis-specific proteins that are destroyed at anaphase I. Furthermore, both associate with meiosis I kinetochores and promote co-orientation using a short STP motif that binds the polo box domain of PLKs. Both also have poorly understood roles in facilitating to a greater or lesser extent the protection of centromeric cohesion and in the case of Spo13 a role in regulating the APC/C. Strikingly, tethering Spo13 to the inner centromere protein Cnp3 largely suppresses the co-orientation defects of *S. pombe moa1* mutants, an effect dependent on Spo13's PBD binding motif [65]. This implies that Moa1 has a function in co-orientation analogous to that of Spo13, namely as a chaperone and activator of PLK at meiosis I kinetochores. Consistent with this, inactivation of PLK in *S. pombe* (Plo1) specifically at kinetochores compromises co-orientation during meiosis I, phenocopying *moa1* mutants. In *S. cerevisiae*, we know that monopolin is a key target of Spo13/Cdc5, though whether monopolin is the kinase's sole target with regard to co-orientation is unclear. In contrast, in *S. pombe* we have little idea as to the target of Moa1/Plo1. If Moa1 and Spo13 really have analogous functions, then former's downstream targets remain to be discovered. Rec8 cohesin is clearly one possibility, but kinetochore proteins might also be.

### Is PLK's involvement in co-orientation universal?

Recently a meiosis-specific protein called meikin sharing certain properties, albeit no sequence homology to either Spo13 or Moa1, has been described in mammals [65]. Both male and female mice lacking meikin are infertile. Though co-orientation and the first meiotic division occur fairly normally, the centromeric cohesion holding dyads together breaks down prematurely, reminiscent of patronus mutants in *A. Thaliana* [66]. Despite co-orientation occurring more or less normally in meikin -/- oocytes, there is a hint that the process is nevertheless compromised. Sister kinetochores are further apart during the process of co-orientation and actually undergo frequent bi-orientation in meikin -/- oocytes lacking chiasmata, a phenotype reminiscent of *moa1* mutants in *S. pombe*. Lastly, meikin like Spo13 and Moa1 disappears after meiosis I, co-precipitates with PLK, and is required for its efficient accumulation at meiosis I kinetochores. Interestingly, meikin also contains an STP motif that might bind PLK's PBD but whether this is important for its function is unclear as it is not well conserved. If all these proteins are meiosis-specific regulators of PLKs at meiosis I kinetochores, along the lines of the founder member Spo13, then a generic name acknowledging their connection with PLKs might be appropriate. Interestingly, like Spo13 patronus contains destruction boxes and may be a regulator as well as a substrate of the APC/C.

## Conclusions and outlook

Genetics started with the Mendel's discovery precisely 150 years ago that germ cells possess both male and female determinants while gametes contain either one or the other but never both. It was chromosome cytology that first shed light on this mystery and the different behavior of centromeres and kinetochores between the first and second meiotic division has been appreciated for a century [67]. Whether the different behavior of centromeres between the first and second meiotic division is due to changes in cell cycle/developmental state of meiosis I and meiosis II cells or due to changes in the state of their chromosomes was first addressed fifty years later by micromanipulation experiments with grasshopper spermatocytes. This revealed that when dyads from meiosis II cells were transferred to meiosis I spindles, they behaved as dyads normally do, namely they bi-oriented and their centromeres disjoined at the first meiotic division. Likewise, when bivalents from meiosis I cells were transferred to meiosis II spindles, they co-oriented and produced dyads at the second meiotic division [68,69]. This implied that the main determinant of chromosome behavior was not the (developmental) state of the cell cycle but rather the state of the chromosome.

The discovery of shugoshins [70,71], the finding that they act by recruiting PP2A [72], characterization of the *S. cerevisiae* monopolin complex, and the finding that cohesin is important for co-orientation in *S. pombe* has revealed some but not all the key chromosomal proteins involved in this process. Unlike co-orientation, which seems to be conferred by diverse mechanisms, the system conferring protection is highly conserved, though interestingly it is not essential in *C.elegans*, possibly because cohesin proximal and distal to chiasmata contains different kleisin subunits and this per se confers protection to the proximal population [73]. The one thing that may be common to co-orientation in both yeasts and mammals is the intimate involvement of Polo-like kinases recruited to meiosis I kinetochores by Spo13-like proteins. Identifying their targets is clearly one of the challenges for the future. How precisely sister kinetochores are prevented from bi-orienting during meiosis I will remain mysterious for some time.

It is hard to say whether knowledge about the molecular mechanisms conferring co-orientation will prove beneficial to humanity from a medical point of view. However, it should be pointed out that mis-segregation of chromosomes during the first meiotic division is responsible for trisomy in humans and trisomy 21 remains by far the most frequent genetic aberration [74]. Most age related infertility in females is thought to arise from chromosome mis-segregation during meiosis. Defects in the complex network of proteins that confer sister chromatid cohesion, its protection, and the co-orientation of sister kinetochores could therefore be of direct relevance to a medical syndrome that along with cancer is one of the characteristics of a modern world in which we grow up more slowly and live longer than our ancestors. Possibly more important in the long term is that greater knowledge of the mechanisms conferring co-orientation may eventually enable us to induce apomixis in plants (the production of diploid asexual seeds) and thereby facilitate the propagation of hybrid plants upon which modern agriculture depends.
